# Joint Testlet Cognitive Diagnosis Modeling for Paired Local Item Dependence in Response Times and Response Accuracy

**DOI:** 10.3389/fpsyg.2018.00607

**Published:** 2018-04-25

**Authors:** Peida Zhan, Manqian Liao, Yufang Bian

**Affiliations:** ^1^Collaborative Innovation Center of Assessment toward Basic Education Quality, Beijing Normal University, Beijing, China; ^2^Measurement, Statistics and Evaluation, Department of Human Development and Quantitative Methodology, University of Maryland, College Park, MD, United States

**Keywords:** cognitive diagnosis models, response time models, response times, local item dependence, testlet, DINA model, PISA

## Abstract

In joint models for item response times (RTs) and response accuracy (RA), local item dependence is composed of local RA dependence and local RT dependence. The two components are usually caused by the same common stimulus and emerge as pairs. Thus, the violation of local item independence in the joint models is called paired local item dependence. To address the issue of paired local item dependence while applying the joint cognitive diagnosis models (CDMs), this study proposed a joint testlet cognitive diagnosis modeling approach. The proposed approach is an extension of Zhan et al. (2017) and it incorporates two types of random testlet effect parameters (one for RA and the other for RTs) to account for paired local item dependence. The model parameters were estimated using the full Bayesian Markov chain Monte Carlo (MCMC) method. The 2015 PISA computer-based mathematics data were analyzed to demonstrate the application of the proposed model. Further, a brief simulation study was conducted to demonstrate the acceptable parameter recovery and the consequence of ignoring paired local item dependence.

## Introduction

Nowadays, it becomes a common practice to collect response time (RT) data as the computer-based tests are applied to large-scale assessments. RT represents the amount of time a respondent spends on an item. It serves as an additional source of information about the working speed of a respondent as well as the time intensity of an item. In the past few decades, a number of studies have been done to model the RTs. Before the year of 2007, the RT modeling studies such as Thissen (1983), Verhelst et al. (1997), and Wang and Hanson (2005) were motivated by the speed-accuracy trade-off (Luce, 1986). However, this trade-off only reflected a within-person relationship between speed and accuracy (van der Linden, 2009) where, given a fixed set of items, a respondent's speed is dependent on his or her accuracy. Therefore, the relationship between speed and accuracy should be modeled at a higher level. To this end, van der Linden (2007) proposed a hierarchical modeling framework to explain the higher-level relationship between speed and accuracy. In this framework, RTs and RA were separately modeled at the first level whereas two correlational structures were modeled at the second level. The correlational structures accounted for either the dependence between person latent speed and latent ability parameters and that between item accuracy-related and item time-related parameters. A comparison study suggested that the hierarchical modeling framework yielded more reasonable outcomes in both real and simulated data than other RT modeling approaches (Suh, 2010). The hierarchical modeling framework was generalized to integrate different measurement models due to its flexible nature (e.g., Klein Entink et al., 2009a,b; Wang et al., 2013; Meng et al., 2015; Molenaar et al., 2015; Wang and Xu, 2015; Fox and Marianti, 2016). However, almost all the previous studies in RT modeling were based on unidimensional item response theory (IRT) models but none used multidimensional measurement models.

Multidimensional tests and cognitive diagnostic assessments become more and more prevalent given the increasing demand for diagnostic test feedback containing refined information. In general, cognitive diagnostic assessments aim at evaluating respondent's mastery status (e.g., mastery or non-mastery) of latent skills or attributes. This information can be provided to teachers or clinicians so that they can determine the remedial instructions or targeted interventions accordingly. Although numerous cognitive diagnosis models (CDMs) have been developed (for review, see Rupp et al., 2010) based on various cognitive and psychological assumptions, almost all of them only utilized information on RA. Recently, Zhan et al. (2017) proposed a joint cognitive diagnosis modeling approach to simultaneously model RTs and RA. In the study of Zhan et al. (2017), the deterministic-inputs, noisy “and” (DINA) model (Macready and Dayton, 1977; Haertel, 1989; Junker and Sijtsma, 2001) and the lognormal RT model (van der Linden, 2006) were used as the measurement models for RA and RTs, respectively. A higher-order latent structure (de la Torre and Douglas, 2004) was introduced to account for the relationship between latent attributes and a continuous higher-order latent ability. Furthermore, a bivariate normal distribution was used to model the relationship between the higher-order latent ability and the latent speed. A similar approach was proposed by Minchen (2017). Unlike Minchen's approach, Zhan et al. (2017)'s approach explicitly modeled the correlation between different item parameters (i.e., within-item characteristic dependency; Fox, 2010; Zhan et al., manuscript submitted for publication) by assuming that they followed a multivariate normal distribution.

A key assumption in the joint models of RA and RTs is local item dependence. Specifically, the observed RA responses are conditionally independent of each other given an individual score in latent ability or a specific latent attribute mastery status, which is denoted as *local RA independence*; in the meanwhile, all the RTs are conditionally independent of each other given the an individual score in latent speed, which is denoted as *local RT independence*. In other words, in the joint models, local item independence is composed of local RA independence and local RT independence, which is known as *paired local item independence*. However, the assumption of local item independence is often violated in educational tests, resulting in local item dependence. One of the most common scenarios that lead to local item dependence is the presence of testlet, where several items are based on a common context (Wainer and Kiely, 1987).

A testlet is defined as a cluster of items that share a common stimulus. The local item dependence resulted from a testlet is called testlet effect. Testlet has been widely adopted in educational tests. For example, in a reading comprehension test, a testlet is formed when a bundle of items are based on the same reading passage. The testlet design makes the assessment process more efficient (DeMars, 2012). While responding to the items within the same testlet, the students only need to process the scenario once and the context information can be applied to all the items in the testlet. However, the testlet design makes it more difficult to measure student's reading ability as the student's performance may be affected by their knowledge or interest in the reading passage content besides their reading ability (Yen, 1993). Thus, item responses within the same testlet may be locally dependent on each other.

Testlet response theory modeling (Wang and Wilson, 2005; Wainer et al., 2007) is one of the most popular approaches to handle testlet effect or local item dependency. As a bi-factor multidimensional IRT model (DeMars, 2006; Li et al., 2006), the testlet response theory model assumes that all the item responses are accounted for by a common factor of latent ability, while the responses within a testlet are further explained by a random testlet effect factor. It has been demonstrated that the presence of testlet effect affects model parameter estimates, equating process, and test reliability estimates (e.g., Sireci et al., 1991; Bradlow et al., 1999; Wang and Wilson, 2005; Wainer et al., 2007; Jiao et al., 2012, 2013; Zhan et al., 2014; Jiao and Zhang, 2015; Tao and Cao, 2016). However, all the studies above only addressed the local RA dependence but none accounted for the local RT dependence.

As aforementioned, the paired local item independence is composed of local RA independence and local RT independence. Given that the item clusters which cause local RA dependence would also result in local RT dependence, and local RA dependence and local RT dependence should emerge in pairs. Thus, the violation of paired local item independence is called *paired local item dependence*. In other words, local RA dependence and its corresponding local RT dependence are caused by the same stimulus but are reflected in different forms (i.e., RA and RTs). To address the paired local item dependence in the IRT framework, Im (2017) proposed a hierarchical testlet model, in which local RA dependence was handled by a testlet response theory model whereas local RT dependence was handled by a lognormal RT testlet model.

In cognitive diagnosis, however, only a few studies focused on accounting for local RA dependence (e.g., Hansen, 2013; Zhan et al., 2015; Hansen et al., 2016), and, to our knowledge, none examined local RT dependence. As aforementioned, the joint CDMs assume paired local item independence. Thus, the purpose of this study is to extend the joint cognitive diagnosis modeling approach (Zhan et al., 2017) in order to address the potential paired local item dependence in RTs and RA. The rest of the paper starts with a review of the testlet-DINA model (Zhan et al., 2015) and the lognormal RT testlet model (Im, 2017). Then the proposed joint testlet-DINA model is introduced. It is followed by a real data analysis using the Program for International Student Assessment (PISA) 2015 computer-based mathematics data, which serves to demonstrate the application of the proposed model. Finally, a brief simulation study is presented used to demonstrate the model parameter recovery and the consequence of ignoring paired local item dependence.

## Joint testlet cognitive diagnosis modeling

### The testlet-DINA model

To account for the local RA dependence in cognitive diagnosis, Hansen (2013) and Hansen et al. (2016) proposed a higher-order, hierarchical CDM which can be viewed as a combination of the two-tier item factor model (Cai, 2010) and the log-linear CDM (Henson et al., 2009). Like the two-tier item factor model, Hansen's model could only account for local RA dependence which was resulted from a single source. Zhan et al. (2015) proposed two within-item multidimensional testlet effect CDMs which was able to account for local RA dependence that was resulted from multiple sources simultaneously (Rijmen, 2011; Zhan et al., 2014). The two models included a compensatory model which allowed attributes to compensate each other and a non-compensatory model which assumed that respondents need to master all the required attributes in order to have a high correct response probability. For simplicity, the testlet-DINA model in this study only refers to the non-compensatory model, which is written as

(1) $$\begin{array}{l} \text{logit}\left(P\left(Y_{ni}=1\right)\right)=\beta_{i}+\delta_{i}\prod_{k=1}^{K}\alpha_{nk}^{q_{ik}}+\sum_{m=1}^{M}u_{im}\gamma_{nm}, \end{array}$$

where *Y*_*ni*_ denotes the dichotomous response of person *n* to item *i*; $\alpha_{n}=(\alpha_{n1},\ldots ,\alpha_{nK})\prime$ denotes person *n*'s attribute pattern, *K* is the number of required attributes; β_*i*_ and δ_*i*_ are the intercept and interaction parameters for item *i*, respectively; The Q-matrix (Tatsuoka, 1983) is an *I*-by-*K* confirmatory matrix with element *q*_*ik*_ indicating whether the attribute *k* is required to correctly answer the item *i* (i.e., *q*_*ik*_ = 1 if the attribute is required, and 0 otherwise); $\gamma_{nm}\sim N\left(0,\sigma_{\gamma_{m}}^{2}\right)$is the RA testlet effect of the *m*th testlet, which represents the interaction effect between person *n* and items within testlet *m* on RA. Usually, the value of $\sigma_{\gamma_{m}}^{2}$indicates the magnitude of testlet effect (Wang and Wilson, 2005; Wainer et al., 2007). A large variance is associated with a large testlet effect. All the γ_*nm*_s are assumed to be independent with each others; Let *M* be the total number of testlets in the test, the U-matrix (Zhan et al., 2014) is an *I*-by-*M* confirmatory matrix with element *u*_*im*_ indicating whether item *i* belongs to testlet *m* (i.e., *u*_*im*_ = 1 if item *i* belongs to testlet *m*, and 0 otherwise).

Obviously, when all elements in the U-matrix equal to 0 (means no tesltet in the test) or all $\sigma_{\gamma_{m}}^{2}=0$ (means no testlet effect), the testlet-DINA model reduces to the reparameterized DINA model (DeCarlo, 2011; von Davier, 2014).

### The lognormal RT testlet model

To account for the local RT dependence, Im (2017) proposed the lognormal RT testlet model. The lognormal RT testlet model is an extension of the regular lognormal RT model (van der Linden, 2006) by introducing a random testlet effect parameter, but it can also be taken as a special case of the multidimensional lognormal RT model (Zhan et al., manuscript submitted for publication). Let *T*_*ni*_ be the observed RT of person *n* to item *i*, the lognormal RT testlet model can be expressed as

(2) $$\begin{array}{l} T_{ni}\sim f\left(t_{ni};\tau_{n},\lambda_{nm},\omega_{i},\xi_{i}\right)\\ =\frac{\omega_{i}}{t_{ni}\sqrt{2\pi }}\exp\left(-\frac{1}{2}{\left(\omega_{i}\left(\log t_{ni}-\left(\xi_{i}-\tau_{n}-\lambda_{nm}\right)\right)\right)}^{2}\right), \end{array}$$

where log*t*_*ni*_ be the logarithm of RT, which is used to transform the positively skewed distribution of RT to a more symmetric shape; τ_*n*_ be the latent speed of person *n*; ξ_*i*_ be the time-intensity of item *i*; ω_*i*_ be the discriminating power of item *i*, which can be treated as a time-kurtosis parameter; $\lambda_{nm}\tilde{\;}N\left(0,\sigma_{\lambda_{m}}^{2}\right)$be the *m*th RT testlet effect parameter to address local RT dependence, which represents the interaction between person *n* and items within testlet *m* in RT. The larger the variance, the larger the testlet effect is. All λ_*nm*_s are assumed to be independent of each other.

Equation (2) can be extended to account for potential within-item multidimensional testlet effect

(3) $$\begin{array}{l} T_{ni}\sim f\left(t_{ni};\tau_{n},{003BB}_{n},\omega_{i},\xi_{i}\right)\\ =\frac{\omega_{i}}{t_{ni}\sqrt{2\pi }}\exp\left(-\frac{1}{2}{\left(\omega_{i}\left(\log t_{ni}-\left(\xi_{i}-\tau_{n}-\sum_{m=1}^{M}u_{im}\lambda_{nm}\right)\right)\right)}^{2}\right), \end{array}$$

where all the parameters have been defined above. Equation (3) is regarded as the within-item multidimensional testlet effect lognormal RT model, which can be seen as a special case of the multidimensional lognormal RT model (Zhan et al., manuscript submitted for publication). For simplicity, Equation (3) can be equivalently expressed as

(4) $$\begin{array}{l} \log T_{ni}\sim N\left(\xi_{i}-\tau_{n}-\sum_{m=1}^{M}u_{im}\lambda_{nm},\omega_{i}^{-2}\right). \end{array}$$

When there is only one source of local RT dependence, the within-item multidimensional testlet effect lognormal RT model reduces to the lognormal RT testlet model (Im, 2017). Further, when all the elements in the U-matrix equal to 0 or $\sigma_{\lambda_{m}}^{2}=0$for all testlets, the within-item multidimensional testlet effect lognormal RT model reduces to the regular lognormal RT model (van der Linden, 2006).

### The joint testlet-DINA model

**Figure 1:**
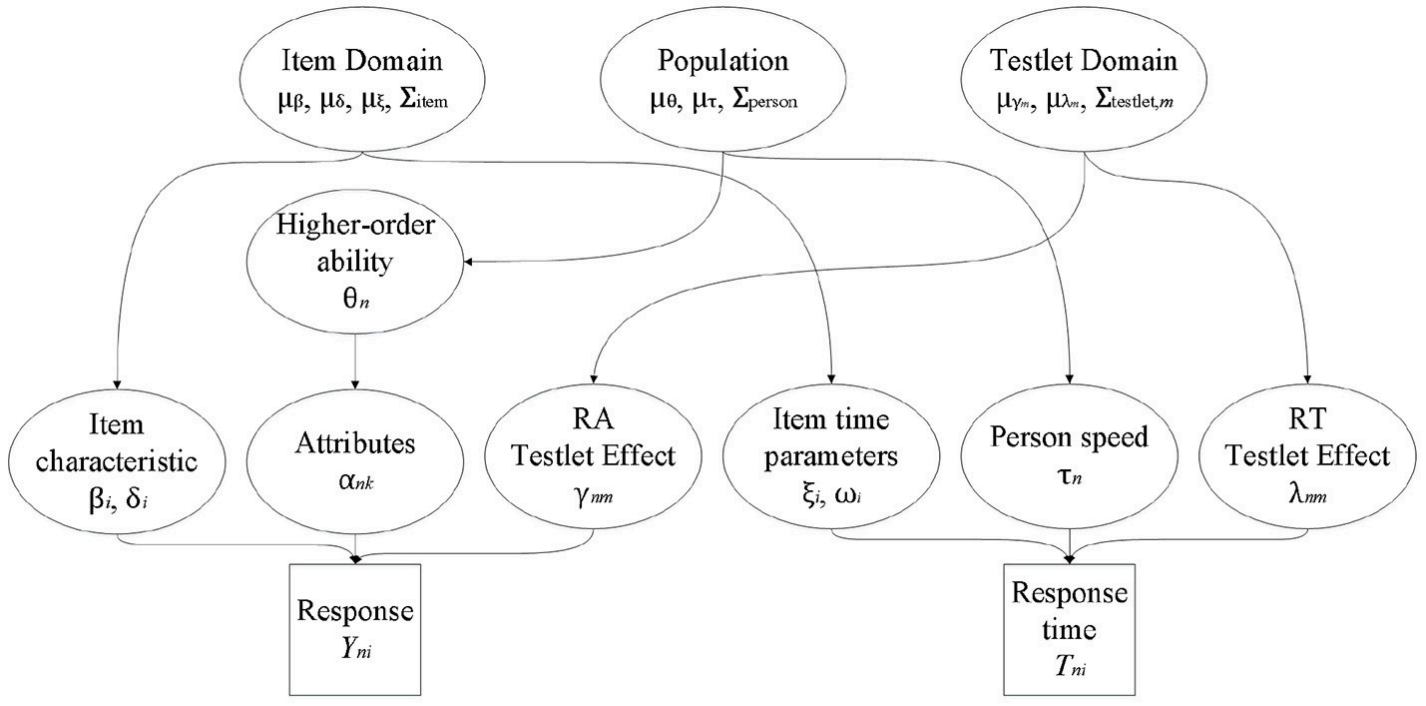
A graphical representation of the joint testlet-DINA model.

The joint testlet-DINA model is specified as follows: *Y*_*ni*_ and log*T*_*ni*_ are separately modeled at the first level following the convention of joint cognitive diagnosis modeling approach and the hierarchical testlet model; a higher-order latent structural model is used to account for the relationship between binary latent attributes and a continuous higher-order latent ability; further, at the higher level, three variance-covariance structures are imposed to model the dependencies among person parameters, item parameters, and testlet effect parameters. A graphical representation of the joint testlet-DINA model is given in Figure 1.

First, the testlet-DINA model (Equation 1) and the within-item multidimensional testlet effect lognormal RT model (Equation 4) are used as the measurement models for RA and RTs, respectively.

Then, the higher-order latent structural model is used to link the correlated attributes, which is given by

(5) $$\begin{array}{l} \text{logit}\left(P\left(\alpha_{nk}=1\right)\right)=\nu_{k}\theta_{n}-\kappa_{k}, \end{array}$$

where *P*(α_*nk*_ = 1) is the probability of mastery of attribute *k* by person *n*; θ_*n*_ is a higher-order (general) ability of person *n*, which is assumed to follow a standard normal distribution for identification purpose; and ν_*k*_ and κ_*k*_ are the slope and difficulty parameters for attribute *k*.

Further, item parameters are assumed to follow a trivariate normal distribution

(6) $$\begin{array}{l} \Psi_{i}=\left(\begin{array}{c} \beta_{i}\\ \delta_{i}\\ \xi_{i} \end{array}\right)\sim N\left(\left(\begin{array}{c} \mu_{\beta }\\ \mu_{\delta }\\ \mu_{\xi } \end{array}\right),\Sigma_{item}\right). \end{array}$$

Additionally, since the residual error variance, $\omega_{i}^{-2}$, is assumed to be independently distributed (Zhan et al., 2017), it is not included in Ψ_*i*_.

Likewise, person parameters are assumed to follow a bivariate normal distribution

(7) $$\begin{array}{l} \Theta_{n}=\left(\begin{array}{c} \theta_{n}\\ \tau_{n} \end{array}\right)\sim N\left(\left(\begin{array}{c} \mu_{\theta }\\ \mu_{\tau } \end{array}\right),\Sigma_{person}\right). \end{array}$$

In addition, testlet effect parameters in testlet *m* are assumed to follow a bivariate normal distribution

(8) $$\begin{array}{l} \Gamma_{nm}=\left(\begin{array}{c} \gamma_{nm}\\ \lambda_{nm} \end{array}\right)\sim N\left(\left(\begin{array}{c} 0\\ 0 \end{array}\right),\Sigma_{testlet,m}\right). \end{array}$$

If there are *M* testlets, there will be *M* bivariate normal distributions. In addition, it should be noted that, in the proposed model, the *u*_*im*_ in RT model (Equation 3) has the same value as the *u*_*im*_ in RA model (Equation 1) because of the paired local item dependence. In summary, Equations (1, 4–8), together, constitute the joint testlet-DINA model. Constraints are set for identification purpose (i.e., $\mu_{\theta }=0,\sigma_{\theta }^{2}=1;\mu_{\tau }=0$). The first two constraints are consistent with those set in the higher-order latent trait model while the third removes the tradeoff between ξ_*i*_ and τ_*n*_ from a lognormal model. After addressing the paired local item dependence, four conditional independence assumptions are made: the α_*nk*_ are conditionally independent given θ_*n*_; the *Y*_*ni*_ are conditionally independent given α_*n*_ and γ_*nm*_; the log*T*_*ni*_ are conditionally independent given τ_*n*_ and λ_*nm*_; and *Y*_*ni*_ and log*T*_*ni*_ for a particular item *i* are conditionally independent given person parameters and testlet effect.

### Bayesian parameter estimation

Parameters in the joint testlet-DINA model can be estimated using the full Bayesian approach with the Markov chain Monte Carlo (MCMC) method. In this study, free software JAGS (Version 4.3.0; Plummer, 2015) was used to estimate the parameters. JAGS uses a default option of the Gibbs sampler (Gelfand and Smith, 1990). Sample code were presented in Appendix. A tutorial of using JAGS for Bayesian CDM estimation can be found in Zhan (2017).

To begin with, under the assumption of local independence, *Y*_*ni*_, log*T*_*ni*_ and α_*nk*_ are independently distributed, which is written as

$$\begin{array}{lll} Y_{ni} & ∼ & \text{Bernoulli}(P(Y_{ni}=1)),\\ \log T_{ni} & ∼ & N(\xi_{i}-\tau_{n}-{\sum^{\text{​}}}_{m=1}^{M}u_{im}\lambda_{nm},\omega_{i}^{-2}),\\ \alpha_{nk} & ∼ & \text{Bernoulli}(P(\alpha_{nk}=1)). \end{array}$$

The priors of item parameters are assumed to be a trivariate normal distribution, written as

(9) $$\begin{array}{l} \left(\begin{array}{c} \beta_{i}\\ \delta_{i}\\ \xi_{i} \end{array}\right)\sim N\left(\left(\begin{array}{c} \mu_{\beta }\\ \mu_{\delta }\\ \mu_{\xi } \end{array}\right),\Sigma_{item}\right),\omega_{i}^{-2}\sim \text{InvGamma}\left(1,1\right). \end{array}$$

Further, the hyper priors are specified as

$$\begin{array}{l} \mu_{\beta }\sim N\left(-2.197,2\right),\\ \mu_{\delta }\sim N\left(4.394,2\right)I\left(\mu_{\delta }>0\right),\\ \mu_{\xi }\sim N\left(3,2\right),\\ \Sigma_{\text{item}}\sim \text{InvWishart}\left({\text{R}}_{\text{item}},3\right), \end{array}$$

where **R**_item_ is a tridimensional identity matrix.

The priors of person parameters are set as

$$\begin{array}{l} \left(\begin{array}{c} \theta_{n}\\ \tau_{n} \end{array}\right)\sim N\left(\left(\begin{array}{c} 0\\ 0 \end{array}\right),\Sigma_{\text{person}}\right). \end{array}$$

As suggested by Zhan et al. (2017), the Cholesky decomposition of the Σ_person_ is used

$$\begin{array}{l} \Sigma_{\text{person}}=\Delta_{\text{person}}\Delta_{\text{person}}^{\prime } \end{array}$$

where

$$\begin{array}{l} \Delta_{\text{person}}=\left(\begin{array}{cc} 1 & 0\\ \varphi & \psi \end{array}\right) \end{array}$$

is a low triangular matrix with positive entries on the diagonal and unrestricted entries below the diagonal; Δ'_person_ is the conjugate transpose of Δ_person_. The priors of the elements in Δ_person_ are specified as φ ~ *N*(0, 1),ψ ~ Gamma(1, 1).

Then, the priors of the higher-order structural parameters are specified as

$$\begin{array}{l} \kappa_{k}\sim N\left(0,4\right),\nu_{k}\sim N\left(0,4\right)I\left(\nu_{k}>0\right). \end{array}$$

In addition, the priors of testlet effect parameters in testlet *m* are specified as

$$\begin{array}{l} \left(\begin{array}{c} \gamma_{nm}\\ \lambda_{nm} \end{array}\right)\sim N\left(\left(\begin{array}{c} 0\\ 0 \end{array}\right),\Sigma_{\text{testlet},\text{m}}\right), \end{array}$$

with the hyper priors of $\Sigma_{\text{testlet},\text{m}}\sim \text{InvWishart}\left({\text{R}}_{\text{testlet},\text{m}},2\right)$, where **R**_testlet,*m*_ is a two-dimensional identity matrix for testlet *m*.

Finally, the posterior mean and the posterior mode are used as the estimates for the continuous parameters (e.g., β_*i*_, δ_*i*_, θ_*n*_, and τ_*n*_) and categorical parameters (e.g., α_*nk*_), respectively.

## Real data analysis

### Data

In this study, the PISA 2015 computer-based mathematics data were used. 17 computer-scored dichotomous items from M1 and M2 testing clusters were selected and used in the analysis. The complete-case method was implemented to handle the missing data. That is, only the respondents without missing values in any of the 17 items were used. As a result, the dataset used for analysis contained the dichotomous response data and continuous RT data for 8,606 respondents from 58 countries/economies. The natural logarithm of RTs (i.e., log RTs) were used for modeling. According to the PISA 2015 mathematics assessment framework (OECD, 2016), 11 attributes were assessed, including change and relationships (α1), space and shape (α2), quantity (α3), uncertainty and data (α4), personal (α5), occupational (α6), societal (α7), scientific (α8), formulating situations mathematically (α9), employing mathematical concepts, facts, procedures and reasoning (α10), and interpreting, and applying and evaluating mathematical outcomes (α11). The first four attributes are associated with the mathematical content knowledge that is targeted for use in the items. The next four attributes are associated with the mathematical context that is needed to place additional demands on the problem-solver (Watson and Callingham, 2003; OECD, 2016). The last three attributes are associated with the mathematical processes that connect the context of the mathematics problem with problem-solving (OECD, 2016). In addition, the 17 items contained four testlets, namely, population pyramids (m1), diving (m2), cash withdrawal (m3), and chair lift (m4). Only one source of local item dependence was considered in this study (i.e., an item only belongs to one testlet). The Q-matrix and the U-matrix are presented in Table 1.

**Table 1:** Q- and U-matrix for PISA 2015 computer-based mathematics items.

**Items**	**Q-matrix**											**U-matrix**			
	**α1**	**α2**	**α3**	**α4**	**α5**	**α6**	**α7**	**α8**	**α9**	**α10**	**α11**	**m1**	**m2**	**m3**	**m4**
CM033Q01		1			1						1				
CM474Q01			1		1					1					
CM155Q01	1							1		1		1			
CM155Q04	1							1			1	1			
CM411Q01			1				1			1			1		
CM411Q02				1			1				1		1		
CM803Q01				1		1			1						
CM442Q02			1				1				1				
CM034Q01		1				1			1						
CM305Q01		1					1			1					
CM496Q01			1				1		1					1	
CM496Q02			1				1			1				1	
CM423Q01				1	1						1				
CM603Q01			1					1		1					
CM571Q01	1							1			1				
CM564Q01			1				1		1						1
CM564Q02				1			1		1						1

Blank means “0.”

### Analysis

In addition to the joint testlet-DINA model, the joint responses and times DINA (denoted as the JRT-DINA) model (Zhan et al., 2017) was also used to fit the data for comparison purpose. The JRT-DINA model can be seen as a special case of the joint testlet-DINA model where all random testlet effect parameters are set to be zero. For both models, two Markov chains with random starting points were used and 10,000 iterations were run for each chain. The first 5,000 iterations in each chain were discarded as burn-in. In order to save space in memory^1^, the thinning interval was set to be five. As a result, 2,000 iterations were retained for model parameter inferences. The potential scale reduction factor (PSRF; Brooks and Gelman, 1998) was computed to assess the convergence of each parameter. PSRF values lower than 1.1 or 1.2 were used as convergence criteria in previous studies (Brooks and Gelman, 1998; de la Torre and Douglas, 2004). In this study, the PSRFs were generally lower than 1.05, indicating good convergence in the specific setting.

The AIC (Akaike, 1974), BIC (Schwarz, 1978), and DIC (Spiegelhalter et al., 2002) were computed for model comparison. Posterior predictive model checking (PPMC; Gelman et al., 2014) was used to evaluate model-data fit. Posterior predictive probability (PPP) values near 0.5 indicate that there are no systematic differences between the observed and predicted values, suggesting an adequate model-data fit. As the research in the absolute model-fit statistics for joint models was limited, this study followed Zhan et al. (2017) to evaluate the model fit of the RA and RT models separately. The sum of the squared Pearson residuals for person *n* and item *i* (Yan et al., 2003) was used as a discrepancy measure to evaluate the overall fit of the RA model, which is written as

$$D\left(Y_{ni};\alpha_{n},\beta_{i},\delta_{i}\right)={\overset{N}{\underset{n=1}{\sum }}\overset{I}{\underset{i=1}{\sum }}\left(\frac{Y_{ni}-P\left(Y_{ni}=1\right)}{\sqrt{P\left(Y_{ni}=1\right)\left(1-P\left(Y_{ni}=1\right)\right)}}\right)}^{2},$$

where *P*(*Y*_*ni*_ = 1) has the same definition as that in Equation (1). On the other hand, the sum of the standardized error function of log*T*_*ni*_ for person *n* and item *i* (Marianti et al., 2014; Fox and Marianti, 2017) was used as a discrepancy measure to evaluate the overall fit of the RT model, which is given by

$$\begin{array}{l} D\left(\log T_{ni};\xi_{i},\tau_{n},\omega_{i}\right)\\ =\overset{N}{\underset{n=1}{\sum }}\overset{I}{\underset{i=1}{\sum }}{\left(\omega_{i}\left(\log T_{ni}-\left(\xi_{i}-\tau_{n}-\sum_{m=1}^{M}u_{im}\lambda_{nm}\right)\right)\right)}^{2}. \end{array}$$

### Results

The joint testlet-DINA model was favored based on the AIC, BIC, and DIC, as is shown in Table 2. In addition, the likelihood deviances (i.e., −2 log likelihood or −2LL) of these two models were 387,466 and 414,438, respectively (Δ −2LL = 26,972, *df* = 12, *p* < 0.001). Therefore, the joint testlet-DINA model fitted the data significantly better than the JRT-DINA model, indicating that paired local item dependence existed among items within testlets. In the joint testlet-DINA model, the PPP values of the RA model and the RT model were 0.486 and 0.547, respectively, which indicated an adequate model-data fit. Thus, only the results pertaining to the joint testlet-DINA model are discussed next (the difference between two models see Figures S1, S2 in Appendix).

**Table 2:** Models fit for PISA 2015 computer-based mathematics.

**Model**	**−2LL**	**AIC**	**BIC**	**DIC**	**NP**	***ppp*_RA**	***ppp*_RT**
Joint testlet-DINA	**387466**	**387648**	**388291**	**525481**	91	0.486	0.547
JRT-DINA	414438	414596	415154	530742	79	0.521	0.539

*−2LL, −2 log-likelihood; AIC, Akaike's information criterion; BIC, Bayesian information criterion; NP, number of parameters; ppp, posterior predictive p-value; RA, item response accuracy; RT, item response time*.

**Figure 2:**
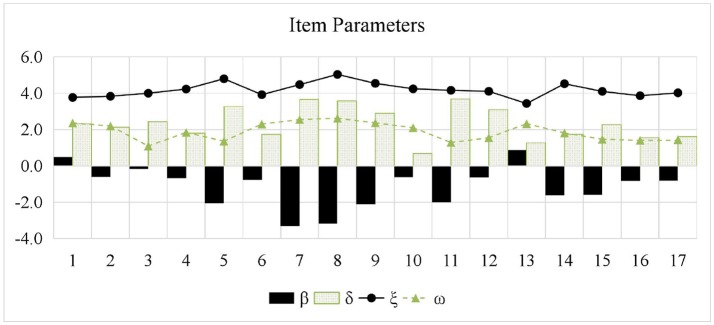
Item parameter estimates for PISA 2015 computer-based mathematics items. β, item intercept; δ, item interaction; ξ, item time-intensity; ω, item time-kurtosis.

Table 3 presents the estimated item mean vector and the estimated item variance-covariance matrix. ρ_βδ_ was estimated to be −0.645, which means that higher item intercept parameters were associated with lower item interaction parameters. ρ_βξ_ and ρ_δξ_ were estimated to be −0.700 and 0.450, respectively, indicating that items with higher intercept parameters tended to have lower time-intensity parameters; by contrast, items with higher interaction parameters tended to be have higher time-intensity parameters. Further, Figure 2 presents the estimated item parameters. All the β_*i*_ estimates were negative except the 1st and the 13th items, which means that the guessing probabilities (i.e.,$\frac{\exp\left(\beta_{i}\right)}{1+\exp\left(\beta_{i}\right)}$) of these two items were higher than 0.5.

**Table 3:** Item mean vector and variance and covariance matrix estimates for PISA 2015 computer-based mathematics items.

	**μ_item_**	**Σ_item_**	**β**	**δ**	**ξ**
μ_β_	−1.232 (0.278)	β	1.436 (0.558)	−0.645	−0.700
μ_δ_	2.394 (0.231)	δ	−0.749 (0.384)	0.938 (0.377)	0.450
μ_ξ_	4.197 (0.113)	ξ	−0.408 (0.198)	0.212 (0.146)	0.236 (0.092)

*Covariance in lower triangular matrix and correlation coefficient in upper triangular matrix, respectively, in Σ_item_; standard error (standard deviation of the posterior distribution) is in parentheses; β, item intercept; δ, item interaction; ξ, item time-intensity*.

Table 4 presents the estimated person variance and covariance matrix. ρ_θτ_ was estimated to be −0.196, which means that a low negative correlation was observed between the higher-order ability and the latent speed parameters. The negative correlation was consistent with the results in Zhan et al. (2017). One reasonable explanation is that low-ability respondents lack motivation in taking the low-stakes test (Wise and Kong, 2005). Thus, the low-ability respondents may have shorter RTs and a greater number of incorrect responses than the high-ability respondents. In addition, the variance of latent speed was quite small (i.e., 0.073), which means the variability in latent speed among all respondents was small.

**Table 4:** Person variance and covariance matrix estimates for PISA 2015 computer-based mathematics items.

**Σ_person_**	**θ**	**τ**
θ	1	−0.196
τ	−0.053 (0.004)	0.073 (0.001)

*Covariance in lower triangular matrix and correlation coefficient in upper triangular matrix, respectively; standard error (standard deviation of the posterior distribution) is in parentheses*.

Table 5 presents the four estimated testlet effect variance-covariance matrices. As aforementioned, a larger variance of testlet effect parameters indicates a larger testlet effect. The variances of the four RA testlet effect parameters were estimated to be 0.438, 0.260, 2.800, and 0.414, respectively. Compared to the variance of the latent trait (i.e., 1.00), the RA testlet effects ranged from small to large^2^. By contrast, the variances of the four RT testlet effect parameters were estimated to be 0.110, 0.083, 0.226, and 0.212, respectively. Although the RT testlet effects were small in terms of the absolute values, their ratios to the variance of latent speed (i.e., 0.073) were around 1.507, 1.137, 3.096, and 2.904, respectively, indicating that the RT testlet effects were large in this dataset. In addition, low correlation was observed between each pair of RA testlet effect and RT testlet effect, indicating that these two types of testlet effects were separable. This is an unexpected result. A moderate or a high correlation was expected since, theoretically speaking, local RA dependence and local RT dependence should be caused by the same stimulus. More practical evidence needs to be accumulated from future studies to explain the results.

**Table 5:** Testlet effect variance and covariance matrix estimates for PISA 2015 computer-based mathematics items.

	**m1: population pyramids**		**m2: diving**		**m3: cash withdrawal**		**m4: chair lift**	
**Σ_testlet_**	**γ**	**λ**	**γ**	**λ**	**γ**	**λ**	**γ**	**λ**
γ	0.438 (0.072)	−0.268	0.260 (0.070)	−0.065	2.800 (0.220)	0.022	0.414 (0.067)	−0.187
λ	−0.059 (0.012)	0.110 (0.007)	−0.010 (0.010)	0.083 (0.005)	0.018 (0.022)	0.226 (0.008)	−0.056 (0.013)	0.212 (0.008)

*Covariance in lower triangular matrix and correlation coefficient in upper triangular matrix, respectively; standard error (standard deviation of the posterior distribution) is in parentheses*.

**Figure 3:**
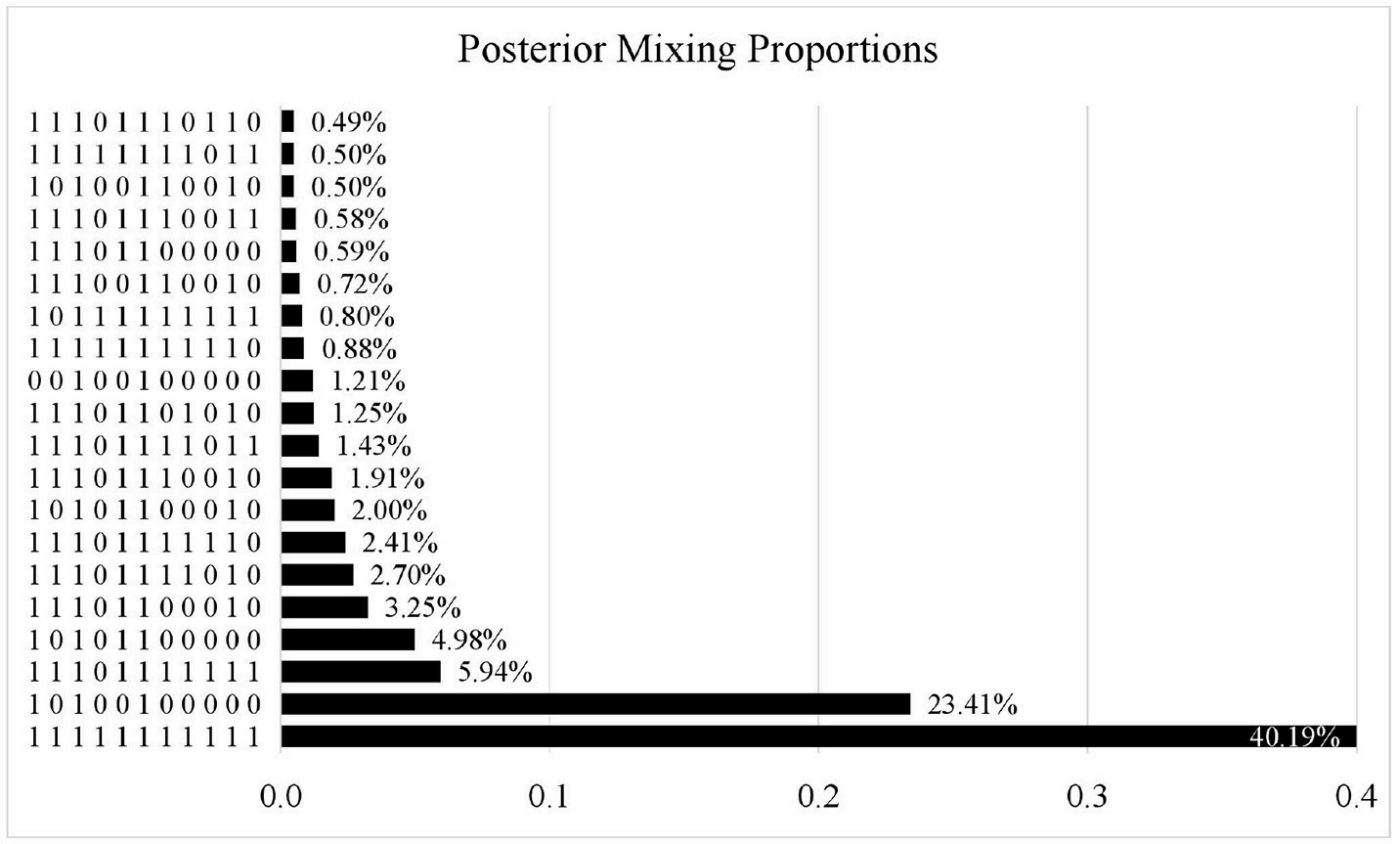
Posterior mixing proportions for PISA 2015 computer-based mathematics items. only the 20 most frequent attribute patterns are displayed.

Figure 3 presents the posterior mixing proportions of the 20 most frequent attribute patterns out of the 2,048 possible attribute patterns. Only 73 patterns were observed in the estimated attribute profiles. Attribute pattern (11111111111) was the most prevalent with a percentage of 40.19%; the second most prevalent pattern was (10100100000) with a percentage of 23.41%.

## A brief simulation study

### Design and data generation

**Figure 4:**
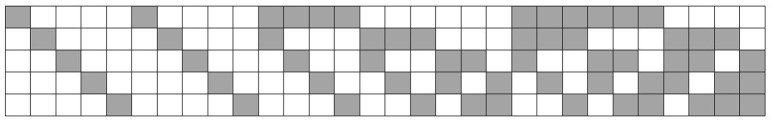
*K*-by-*I* Q' matrix for simulation study. blank means “0,” gray means “1”.

A brief simulation study was conducted to examine the parameter recovery of the proposed model and the consequence of ignoring the potential paired local item dependence in analysis. The simulated dataset contained 1,000 respondents and 30 items measuring five attributes. The Q-matrix is presented in Figure 4. The last 20 items were evenly divided into 4 testlets. Specifically, testlet 1 consisted of items 11 ~ 15, testlet 2 consisted of items 16 ~ 20, testlet 3 consisted of items 21 ~ 25, and testlet 4 consisted of items 26 ~ 30. For simplicity, the four pairs of RA and RT testlet effects were generated from a same bivariate normal distribution,

$$\left(\begin{array}{c} \gamma_{nm}\\ \lambda_{nm} \end{array}\right)\sim N\left(\left(\begin{array}{c} 0\\ 0 \end{array}\right),\left(\begin{array}{cc} 0.50\\ -0.25 & 0.50 \end{array}\right)\right),$$

where ρ_γλ_ = −0.5. Typically, setting the testlet effect as 0.5 indicates a moderate testlet effect (Wang and Wilson, 2005; Wainer et al., 2007). In addition, each item was assumed to belong to only one testlet. Item parameters were generated from a trivariate normal distribution,

$$\left(\begin{array}{c} \beta_{i}\\ \delta_{i}\\ \xi_{i} \end{array}\right)\sim N\left(\left(\begin{array}{c} -2.197\\ 4.394\\ 4.000 \end{array}\right),\left(\begin{array}{ccc} 1.00\\ -0.80 & 1.00\\ -0.25 & 0.15 & 0.25 \end{array}\right)\right),$$

where ρ_βδ_ = −0.8, ρ_βξ_ = −0.5, and ρ_δξ_ = 0.3, which were set according to the estimates from the real data analysis (Zhan et al., 2017); ω_*i*_ were generated from *N*(2, 0.25). Person parameters were generated from a bivariate normal distribution,

$$\left(\begin{array}{c} \theta_{n}\\ \tau_{n} \end{array}\right)\sim N\left(\left(\begin{array}{c} 0\\ 0 \end{array}\right),\left(\begin{array}{cc} 1.00\\ -0.25 & 0.25 \end{array}\right)\right),$$

where ρ_θτ_ = −0.5. For higher-order structural parameters, ν_*k*_ = 1.5 for all the attributes and κ_*k*_ = (−1.0, −0.5, 0.0, 0.5, 1.0), indicating moderate correlations among attributes. The mastery status of each person on each attribute was generated from a Bernoulli distribution with the parameter, *P*(α_*nk*_ = 1) which was computed based on Equation (5).

### Analysis

Thirty replications were implemented. Both the joint testlet-DINA model and the JRT-DINA model were fit to the simulated data. In each replication, the number of chains, burn-in iterations, and post-burn-in iterations were consistent with those in the real data analysis. Convergence was well achieved (see Figure S3 in Appendix). The bias and root mean square error (RMSE) were used to evaluate parameter recovery, which were calculated as $bias\left(\hat{\upsilon }\right)=\sum_{r=1}^{R}\frac{{\hat{\upsilon }}_{r}-\upsilon }{R}$ and $RMSE\left(\hat{\upsilon }\right)=\sqrt{\sum_{r=1}^{R}\frac{{\left({\hat{\upsilon }}_{r}-\upsilon \right)}^{2}}{R}}$, where $\hat{\upsilon }$ and υ are the estimated and true value of model parameters, respectively; *R* is the number of replications. In addition, the correlation between the true and estimated value of model parameters was computed. In terms of the classification accuracy, the attribute correct classification rate (ACCR) and pattern correct classification rate (PCCR) were computed as $\text{ACCR}=\frac{\overset{R}{\underset{r=1}{\sum }}\overset{N}{\underset{n=1}{\sum }}W_{nk}}{R\times N}$ and $\text{PCCR}=\frac{\sum_{r=1}^{R}\sum_{n=1}^{N}\prod_{k=1}^{K}W_{nk}}{R\times N}$, where *W*_*nk*_ = 1 if $\alpha_{nk}={\hat{\alpha }}_{nk}$, and *W*_*nk*_ = 0 otherwise.

### Results

In all the 30 replications, the joint tesltet-DINA model was favored by AIC, BIC and DIC, which indicates that the three fit indices can select the best-fitting model correctly.

**Figure 5:**
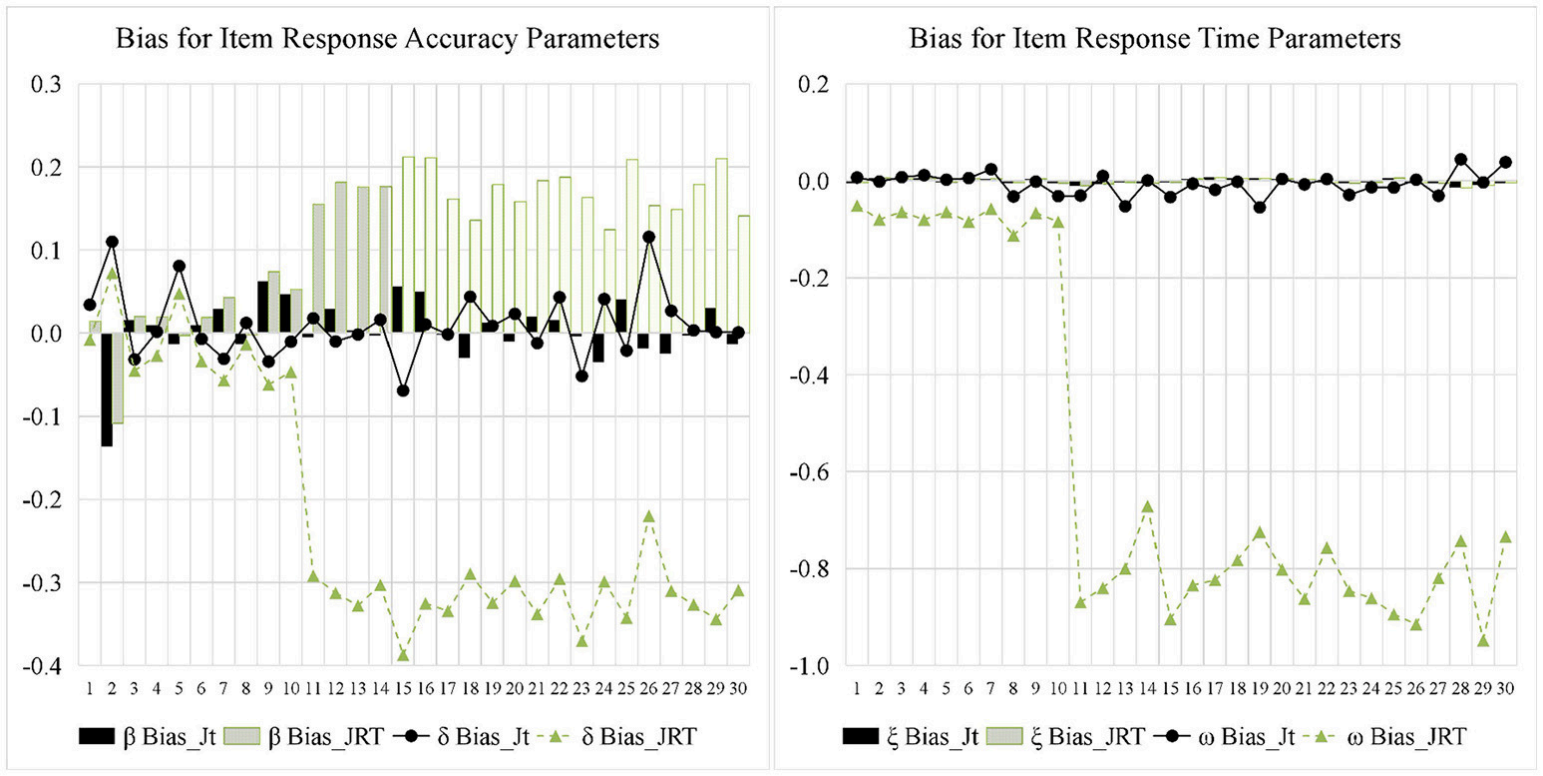
Bias for item parameter in simulation study. Jt, joint testlet-DINA model; JRT, JRT-DINA model; β, item intercept; δ, item interaction; ξ, item time-intensity; ω, item time-kurtosis.

**Figure 6:**
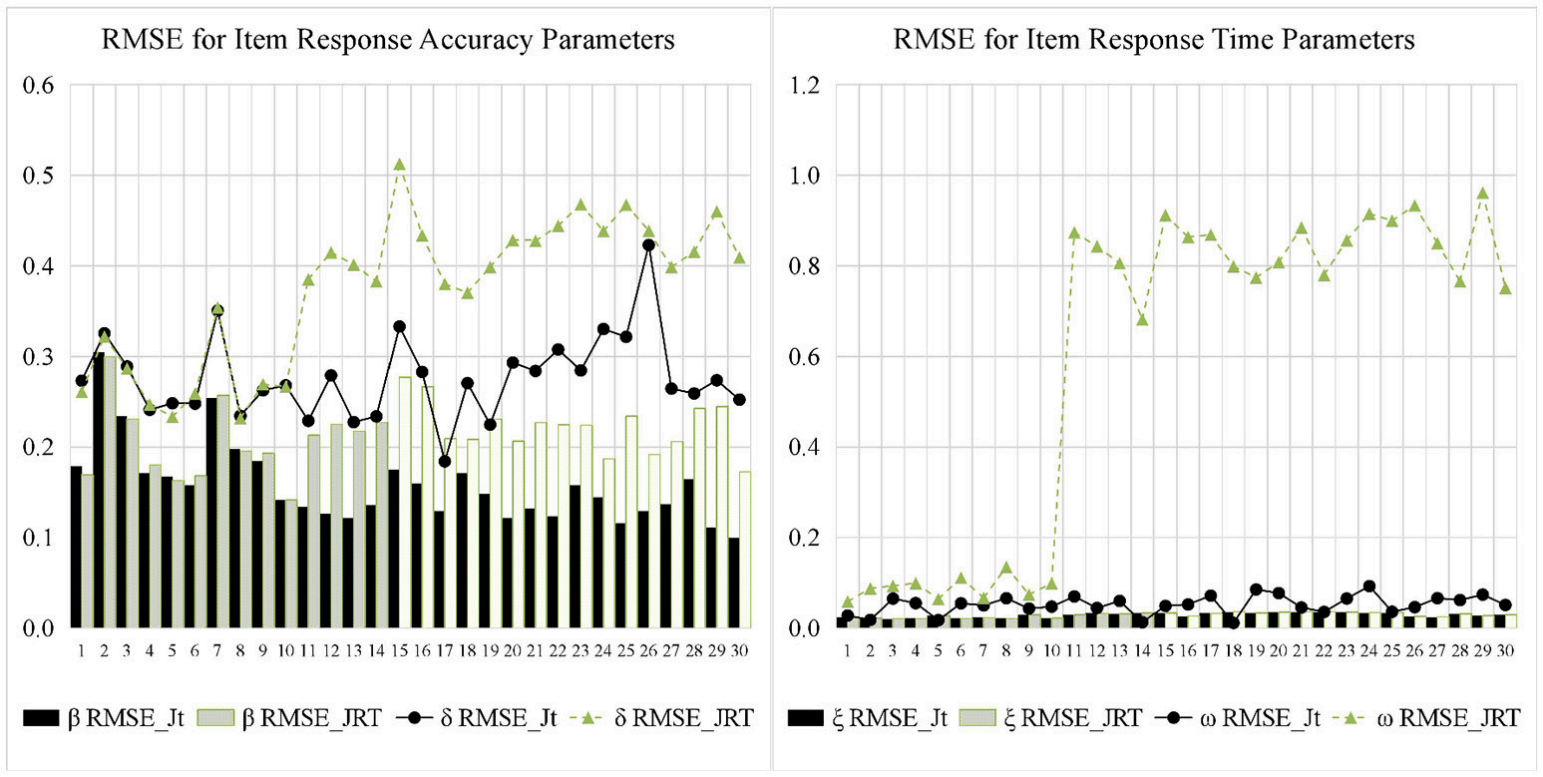
Root mean square error (RMSE) for item parameter in simulation study. Jt, joint testlet-DINA model; JRT, JRT-DINA model; β, item intercept; δ, item interaction; ξ, item time-intensity; ω, item time-kurtosis.

Figures 5, 6 display the recovery of the item parameters for the two models. According to the results of the last 20 items with testlet structure, the performance of the JRT-DINA model was significantly affected by the paired local item dependence. Specifically, ignoring paired local item dependence in analysis would result in overestimation of item intercept parameters, underestimation of item interaction parameters, and underestimation of item time-kurtosis parameters. However, it had little effect on the recovery of item time-intensity parameters. In addition, most of the 10 items without testlet structure had smaller absolute bias in parameter estimates from the joint testlet-DINA model than from the JRT-DINA model; the RMSE of the parameter estimates from the joint testlet-DINA model was equal to or smaller than those from the JRT-DINA model. Table 6 further summarizes the item parameter recovery by presenting the mean absolute bias, the mean RMSE, and the correlation between estimated and true values of all the items. Again, it can be seen that ignoring the paired local item dependence mainly affected the recovery of item time-kurtosis parameters. In addition, the item RT parameters were recovered better than the item RA parameters in joint models.

**Table 6:** Summary of the item parameter recovery in simulation study.

**Index**	**Item parameter**	**Joint testlet-DINA**	**JRT-DINA**
MA_Bias	β	0.025	0.127
	δ	0.029	0.225
	ξ	0.004	0.004
	ω	**0.017**	**0.572**
M_RMSE	β	0.158	0.214
	δ	0.277	0.374
	ξ	0.029	0.029
	ω	**0.052**	**0.591**
Correlation	β	0.986	0.982
	δ	0.958	0.946
	ξ	0.999	0.999
	ω	**0.973**	**0.123**

*MA_Bias, mean absolute value of bias across all items; M_RMSE, mean value of root mean square error across all items; Correlation, correlation between estimated and true values of all items; β, item intercept; δ, item interaction; ξ, item time-intensity; ω, item time-kurtosis; JRT-DINA, joint responses and times DINA model*.

**Figure 7:**
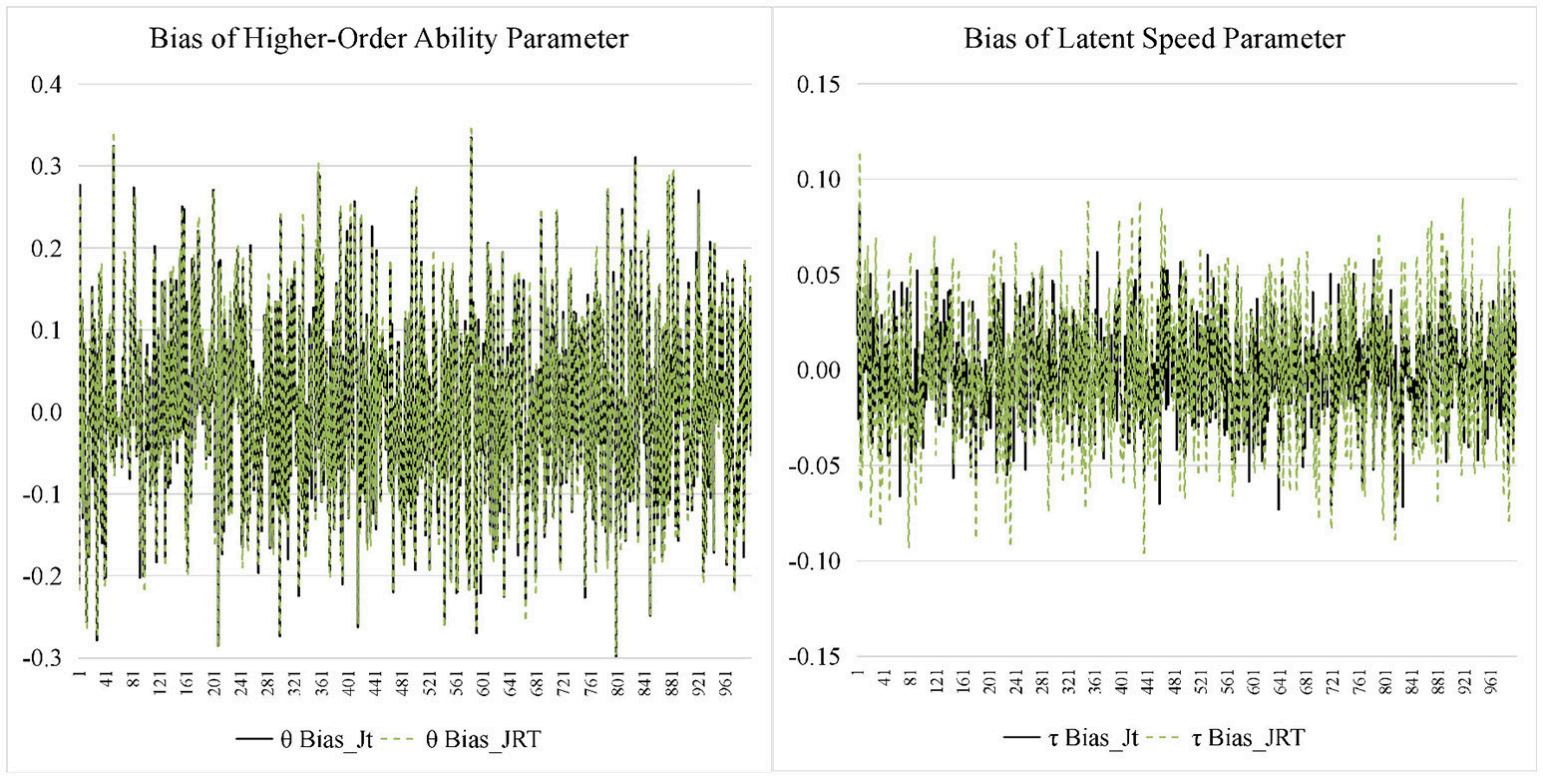
Bias for person parameter in simulation study. Jt, joint testlet-DINA model; JRT, JRT-DINA model; θ, higher-order latent ability; τ, latent speed.

**Figure 8:**
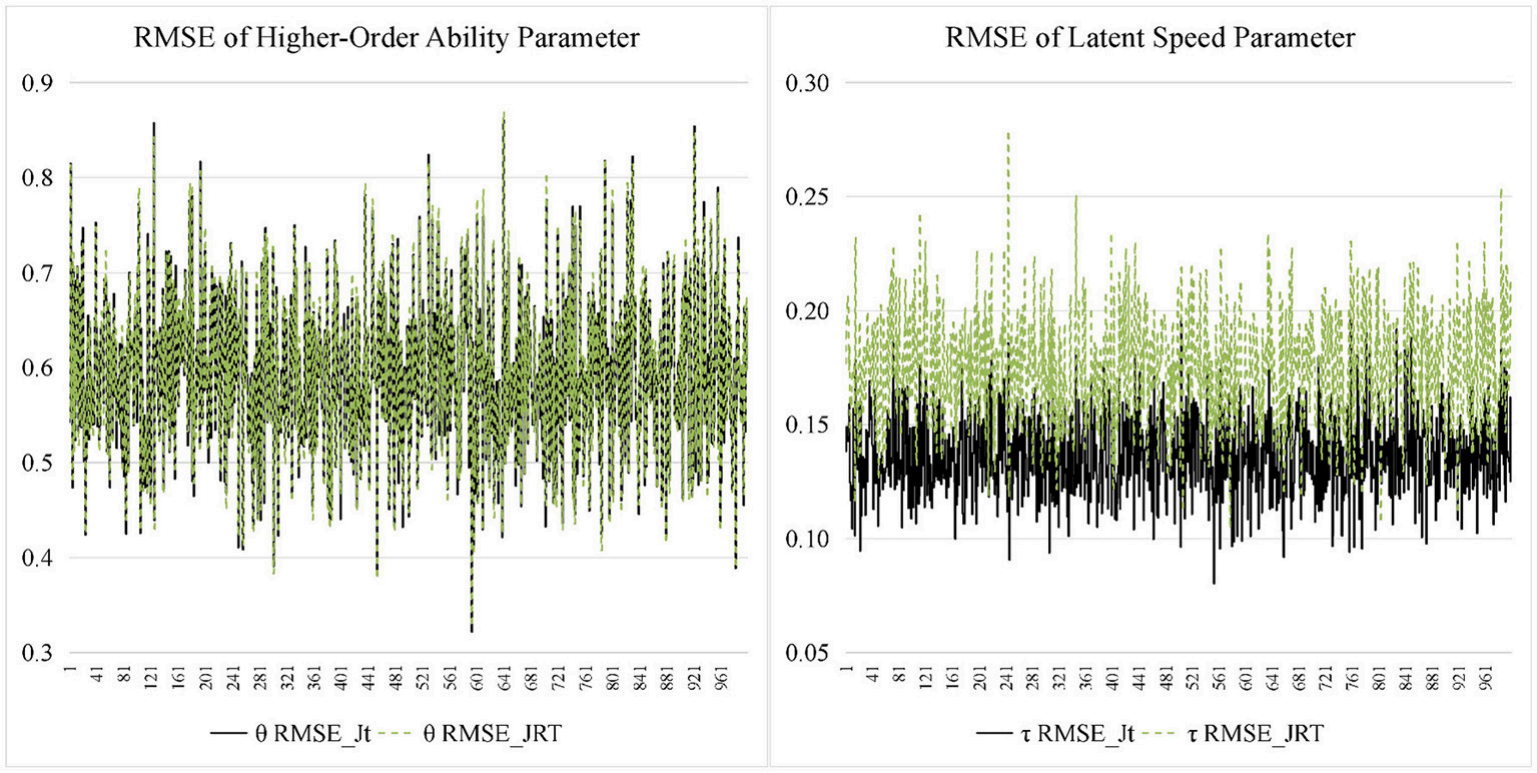
Root mean square error (RMSE) for person parameter in simulation study. Jt, joint testlet-DINA model; JRT, JRT-DINA model; θ, higher-order latent ability; τ, latent speed.

Figures 7, 8 display the recovery of the person parameters for the two models. The two models performed similarly on recovering the higher-order ability parameter. In terms of the latent speed parameters, the bias was similar for the two models, but the RMSE from the JRT-DINA model was significantly larger than that from the joint testlet-DINA model. The results indicate that ignoring the paired local item dependence in analysis would result in large variability in latent speed parameters but had little effect on the recovery of higher-order ability parameters. Table 7 further summarizes the recovery of person parameters. The two models mainly differed in the mean RMSE of latent speed across person. In addition, the recovery of latent speed parameters was better than that of the higher-order ability parameters.

**Table 7:** Summary of the person parameter recovery in simulation Study.

**Index**	**Item parameter**	**Joint testlet-DINA**	**JRT-DINA**
MA_Bias	θ	0.088	0.088
	τ	0.020	0.026
M_RMSE	θ	0.593	0.595
	τ	**0.137**	**0.175**
Cor	θ	0.803	0.801
	τ	0.961	0.939

*MA_Bias, mean absolute value of bias across all persons; M_RMSE, mean value of root mean square error across all persons; Correlation, correlation between estimated and true values of all persons; θ, higher-order latent ability; τ, latent speed*.

Table 8 presents the recovery of individual attributes and attribute patterns. The joint testlet-DINA model was higher than the JRT-DINA model in both ACCR and PCCR, which indicates that ignoring the paired local item dependence would slightly reduce attribute and pattern correct classification rates (PCCRs).

**Table 8:** Attribute and pattern correct classification rate in simulation study.

**Analysis model**	**ACCR**					**PCCR**
	**α1**	**α2**	**α3**	**α4**	**α5**	
Joint testlet-DINA	0.974	0.961	0.968	0.973	0.980	0.872
JRT-DINA	0.974	0.961	0.967	0.973	0.979	0.870

*ACCR, attribute correct classification rate; PCCR, pattern correct classification rate*.

Table 9 presents the recovery of item, person and testlet variance-covariance matrices. First, in terms of the item variance-covariance matrix, the bias was similar for the two models, but the RMSE from the joint testlet-DINA model was larger than that from the JRT-DINA model. Second, the latent speed variance was recovered better in the joint testlet-DINA model than in the JRT-DINA model. Third, all the four testlet variance-covariance matrices were well recovered. The recovery of the RT testlet effect variance parameters was better than that of the RA testlet effect variance parameters.

**Table 9:** Recovery of Variance and Covariance Matrices in Simulation Study.

**Parameter**			**Joint testlet-DINA**		**JRT-DINA**	
			**Bias**	**RMSE**	**Bias**	**RMSE**
Σ_item_	Variance of intercept	$\sigma_{\text{β}}^{2}$	0.043	0.230	−0.032	0.208
	Covariance of intercept and interaction	σ_βδ_	−0.001	0.211	0.057	0.202
	Covariance of intercept and time-intensity	σ_βξ_	−0.008	0.096	0.004	0.089
	Variance of interaction	$\sigma_{\text{δ}}^{2}$	0.035	0.253	−0.033	0.242
	Covariance of interaction and time-intensity	σ_δξ_	0.007	0.099	−0.004	0.089
	Variance of time-intensity	$\sigma_{\text{ξ}}^{2}$	0.062	0.088	0.062	0.088
Σ_person_	Covariance of ability and speed	σ_θτ_	0.004	0.020	0.003	0.021
	Variance of speed	$\sigma_{\text{τ}}^{2}$	−0.001	0.010	0.019	0.022
Σ_testlet,1_	Variance of 1st RA testlet effect	$\sigma_{\text{γ}1}^{2}$	−0.010	0.098		
	Covariance of 1st pair of testlet effects	σ_γ1λ1_	0.008	0.048		
	Variance of 1st RT testlet effect	$\sigma_{\text{λ}1}^{2}$	0.002	0.032		
Σ_testlet,2_	Variance of 2nd RA testlet effect	$\sigma_{\text{γ}2}^{2}$	0.013	0.104		
	Covariance of 2ndt pair of testlet effects	σ_γ2λ2_	0.000	0.038		
	Variance of 2nd RT testlet effect	$\sigma_{\text{λ}2}^{2}$	0.009	0.028		
Σ_testlet,3_	Variance of 3rd RA testlet effect	$\sigma_{\text{γ}3}^{2}$	0.005	0.108		
	Covariance of 3rd pair of testlet effects	σ_γ3λ3_	0.009	0.034		
	Variance of 3rd RT testlet effect	$\sigma_{\text{λ}3}^{2}$	0.006	0.025		
Σ_testlet,4_	Variance of 4th RA testlet effect	$\sigma_{\text{γ}4}^{2}$	0.014	0.107		
	Covariance of 4th pair of testlet effects	σ_γ4λ4_	−0.003	0.041		
	Variance of 4th RT testlet effect	$\sigma_{\text{λ}4}^{2}$	0.008	0.028		

*RMSE, root mean square error*.

Table 10 presents the recovery of item mean vector components and higher-order structural parameters. The item mean vector component estimates from the joint testlet-DINA model had smaller absolute bias and RMSE than those from the JRT-DINA model. The two models performed similarly on recovering the higher-order structural parameters. The results indicate that ignoring the paired local item dependence in analysis would result in less precise item mean vector component estimates, but had little effect on the higher-order structural parameter recovery.

**Table 10:** Recovery of item mean vector and higher-order structural parameters.

**Parameter**			**Joint testlet-DINA**		**JRT-DINA**	
			**Bias**	**RMSE**	**Bias**	**RMSE**
μ_item_	Mean intercept	μ_β_	−0.001	0.178	0.110	0.204
	Mean interaction	μ_δ_	−0.006	0.214	−0.229	0.311
	Mean time-intensity	μ_ξ_	0.012	0.094	0.013	0.095
κ	Difficulty of attribute 1	κ_1_	0.008	0.114	0.008	0.119
	Difficulty of attribute 2	κ_2_	0.002	0.108	0.002	0.107
	Difficulty of attribute 3	κ_3_	−0.010	0.111	−0.010	0.111
	Difficulty of attribute 4	κ_4_	0.048	0.121	0.048	0.119
	Difficulty of attribute 5	κ_5_	0.007	0.103	0.002	0.099
ν	Slope of attribute 1	ν_1_	−0.006	0.151	−0.006	0.150
	Slope of attribute 2	ν_2_	0.049	0.191	0.052	0.198
	Slope of attribute 3	ν_3_	−0.007	0.190	−0.008	0.189
	Slope of attribute 4	ν_4_	0.105	0.230	0.106	0.227
	Slope of attribute 5	ν_5_	−0.056	0.170	−0.060	0.168

*RMSE, root mean square error*.

Overall, the model parameters of the joint testlet-DINA model were well recovered by using the proposed MCMC estimation algorithm. Additionally, ignoring the paired local item dependence in analysis would result in biased model parameter estimates and lower correct classification rates. Specifically, it would result in overestimation of item intercept parameters, underestimation of item interaction parameters, and underestimation of item time-kurtosis parameters. It would lead to less precise estimates of latent speed parameters and item mean vector components. It would also reduce attribute and PCCRs. However, it had little effect on the recovery of item time-intensity parameters, the higher-order ability parameters, or the higher-order structural parameters.

## Conclusion and discussion

To address the paired local item dependence in RT and RA when applying the joint CDMs, this study proposed a joint testlet cognitive diagnosis modeling approach. As an extension of the joint cognitive diagnosis modeling approach (Zhan et al., 2017), the proposed approach modeled the relationship between each pair of RA testlet effect and RT testlet effect using correlational structure. Specifically, the testlet-DINA model and the within-item multidimensional testlet effects lognormal RT model were adopted as the RA model and RT model, respectively. The model parameters were estimated using the full Bayesian MCMC method. The 2015 PISA computer-based mathematics data were analyzed to demonstrate the application of the proposed model. The real data analysis results are summarized as follows: (a) a negative correlation was observed between the higher-order ability and latent speed; (b) a negative correlation was observed between the item intercept parameters and the item time-intensity parameters; (c) a positive correlation was observed between the item interaction parameters and the item time-intensity parameters; (d) the magnitude of RA testlet effects varied from small to large whereas the magnitude of RT testlet effects was large; and (e) low correlation coefficients between the RA and RT testlet effects were found. Overall, most results in this real data analysis were consistent with those in Zhan et al. (2017) that used PISA 2012 computer-based mathematics data. Further, a simulation study was conducted to examine model parameter recovery of the proposed model and the consequence of ignoring testlet effects. The results indicated that the model parameters of the proposed model can be well recovered. Additionally, ignoring the paired local item dependence in analysis would result in biased model parameter estimates and low individual correct classification rates.

Despite the promising results, further research is needed. First, only a DINA-based testlet model and a lognormal RT-based testlet model were used for illustration in this study. In the future study, other CDMs (e.g., von Davier, 2008; Henson et al., 2009; de la Torre, 2011) and RT models (e.g., Klein Entink et al., 2009b; Wang et al., 2013) can be used as the measurement models of RA and RTs. Second, in this study, the proposed model was evaluated using a brief simulation where only a limited number of factors were manipulated. More factors (e.g., test length, number of attributes, magnitude of testlet effects, etc.) and replications are recommended in future studies. Third, the model-data fit of RA and RT models was evaluated separately because of the lack of model-data fit indices for the joint models. In the future studies, absolutely model-fit indices designed for joint models can be explored and further be applied to evaluate the current modeling approach. Fourth, in educational and psychological measurements, latent speed can be defined as the ratio of the amount of labor spent on the items with respect to time (van der Linden, 2011). Due to the multidimensional nature of labors, latent speed may also be a multidimensional concept, each dimension of which corresponds to a specific type of labor. The latent speed was treated as a unidimensional latent trait in this study although the RT testlet effect can be regarded as a specific factor that is relevant to the working speed. Recently, Zhan et al., Manuscript submitted for publication proposed a multidimensional lognormal RT model to account for the potential multidimensionality of latent speed. One possible extension of the current joint modeling approach is to account for the multidimensional latent speed. Fifth, as noted by one of the anonymous reviewers, if there are many testlets, there will be many bivariate covariance matrices to be estimated, leading to large computational burden. Further exploration is needed to deal with this challenging issue. Sixth, in this study, respondents were assumed to be from the same population group, but, in reality, they may be from different groups (e.g., male and female). Multiple group joint modeling (e.g., Jiao et al., 2017) and mixture modeling (e.g., von Davier, 2008) can be incorporated into the current modeling approach in the future. Seventh, in practice, students are nested within classrooms, and classrooms are further nested within schools. Thus, multilevel modeling (e.g., Fox and Glas, 2001; Jiao et al., 2012; Jiao and Zhang, 2015) extension can also be a future direction. Finally, the generalizability of the results from this study is limited given that only data from a low-stakes test were analyzed. More empirical studies based on data from other tests, especially high-stakes tests, are needed.

## Author contributions

PZ: Design of the study, data analysis, paper writing, and revision; ML: Data cleaning, interpretation of data for the work, and paper revision; YB: Preliminary idea construction, paper revision, and proofreading.

### Conflict of interest statement

The authors declare that the research was conducted in the absence of any commercial or financial relationships that could be construed as a potential conflict of interest.

## Footnotes

^1^All calculations were conducted on a laptop with 32GB of memory. Insufficient space of memory was caused by no thinning.

^2^According to previous studies (e.g., Wainer and Wang, 2000; Wang and Wilson, 2005), the value of 0.25, 0.5, and 1.0 is corresponding to small, moderate, and large testlet effect, respectively.
