# Supplementary material for: Joint Testlet Cognitive Diagnosis Modeling for Paired Local Item Dependence in Response Times and Response Accuracy
Source: Front Psychol. 2018 Apr 25;9:607. doi: 10.3389/fpsyg.2018.00607 (PMC5996944; doi:10.3389/fpsyg.2018.00607)
Supplement: Supplementary file 1 [file Data_Sheet_1.docx]

**Appendix:**

**JAG code for the joint testlet-DINA model**

#N: sample size

#I: test length

#K: number of attributes

#attribute: latent attribute

#Q: Q-matrix

#Score: item response accuracy data

#logT: log item response time data

#beta: item intercept parameter

#delta: item interaction parameter

#yita: item time-intensity parameter

#omiga: the square of the item time-kurtosis parameter

#d: the testlet identifier vector. The element in vector d is used to specify item i is part of which testlet. See the tutorial by Zhan (2017).

#theta[,1]: higher-order ability

#theta[,2]: latent speed

#gamma: local RA dependence

#lamda: local RT dependence

#lamda_0: attribute difficulty parameter

#lamda_K: attribute slope parameter

#Sigma_theta: person variance and covariance matrix

#Sigma_item: item variance and covariance matrix

#Sigma_testlet_1: the first testlet variance and covariance matrix

model

{

#measurment model for RA and RT

for (n in 1:N) {

for (i in 1:I) {

for (k in 1:K) {w[n, i, k] <- pow(attribute[n, k], Q[i, k])}

logit(prob[n, i]) <- beta[i] + delta[i] * prod(w[n, i, ]) + gamma[n, d[i]]

Score[n, i] ~ dbern(prob[n, i])

logT[n, i] ~ dnorm(yita[i] - theta[n, 2] - lamda[n, d[i]], omiga[i])

}}

#higher-order latent structural model

for (n in 1:N) {

for (k in 1:K) {

logit(att_prob[n, k]) <- lamda_K[k] * theta[n, 1] - lamda_0[k]

attribute[n, k] ~ dbern(att_prob[n, k])

}}

#prior of person parameters

for (n in 1:N) {theta[n, 1:2] ~ dmnorm(person_mu[1:2], person_cov[1:2, 1:2])}

#prior of item parameters

for (i in 1:I) {

itemparameter[i, 1:3] ~ dmnorm(item_mu[1:3], item_cov[1:3, 1:3])

beta[i] <- itemparameter[i, 1]

delta[i] <- itemparameter[i, 2]

yita[i] <- itemparameter[i, 3]

omiga[i] ~ dgamma(1, 1)}

#prior of testlet parameters

for (n in 1:N){

testlet_1[n, 1:2] ~ dmnorm(testlet_mu[1:2], testlet_cov_1[1:2, 1:2])

testlet_2[n, 1:2] ~ dmnorm(testlet_mu[1:2], testlet_cov_2[1:2, 1:2])

testlet_3[n, 1:2] ~ dmnorm(testlet_mu[1:2], testlet_cov_3[1:2, 1:2])

testlet_4[n, 1:2] ~ dmnorm(testlet_mu[1:2], testlet_cov_4[1:2, 1:2])

gamma[n,1] <- testlet_1[n,1]

lamda[n,1] <- testlet_1[n,2]

gamma[n,2] <- testlet_2[n,1]

lamda[n,2] <- testlet_2[n,2]

gamma[n,3] <- testlet_3[n,1]

lamda[n,3] <- testlet_3[n,2]

gamma[n,4] <- testlet_4[n,1]

lamda[n,4] <- testlet_4[n,2]

gamma[n,5] <- 0

lamda[n,5] <- 0}

#prior of latent structual parameters

for (k in 1:K) {

lamda_0[k] ~ dnorm(0, 0.25)

lamda_K[k] ~ dnorm(0, 0.25) T(0, )}

#hyper priors

person_mu[1]<-0

person_mu[2]<-0

testlet_mu[1]<-0

testlet_mu[2]<-0

L_theta[1, 1] <- 1

L_theta[2, 2] ~ dgamma(1, 1)

L_theta[2, 1] ~ dnorm(0,1)

L_theta[1, 2] <- 0

Sigma_theta <- L_theta %*% t(L_theta)

person_cov[1:2, 1:2] <- inverse(Sigma_theta[1:2, 1:2])

item_mu[1] ~ dnorm(-2.197, 0.5)

item_mu[2] ~ dnorm(4.394, 0.5)

item_mu[3] ~ dnorm(3,0.5)

R_item[1, 1] <- 1

R_item[2, 2] <- 1

R_item[3, 3] <- 1

R_item[1, 2] <- 0

R_item[1, 3] <- 0

R_item[2, 1] <- 0

R_item[2, 3] <- 0

R_item[3, 1] <- 0

R_item[3, 2] <- 0

item_cov[1:3, 1:3] ~ dwish(R_item[1:3, 1:3], 3)

Sigma_item[1:3, 1:3] <- inverse(item_cov[1:3, 1:3])

R_testlet[1, 1] <- 1

R_testlet[2, 2] <- 1

R_testlet[1, 2] <- 0

R_testlet[2, 1] <- 0

testlet_cov_1[1:2, 1:2] ~ dwish(R_testlet[1:2,1:2],2)

testlet_cov_2[1:2, 1:2] ~ dwish(R_testlet[1:2,1:2],2)

testlet_cov_3[1:2, 1:2] ~ dwish(R_testlet[1:2,1:2],2)

testlet_cov_4[1:2, 1:2] ~ dwish(R_testlet[1:2,1:2],2)

Sigma_testlet_1[1:2,1:2] <- inverse(testlet_cov_1)

Sigma_testlet_2[1:2,1:2] <- inverse(testlet_cov_2)

Sigma_testlet_3[1:2,1:2] <- inverse(testlet_cov_3)

Sigma_testlet_4[1:2,1:2] <- inverse(testlet_cov_4)}

**Figure S1**. Item parameter estimates of the JRT-DINA model and the joint testelt-DINA model in the empirical example.

*Note*, testlet items in blue background.

**Figure S2**. Difference in person parameter estimates of the JRT-DINA model and the joint testlet-DINA model in the empirical example.

*Note*, the value equals to the estimate of the joint testlet-DINA model minus that of the JRT-DINA model.

**Figure S3**. Potential scale reduction factor for monitored parameters in simulation study.

*Note*: for the JRT-DINA model, PSRF assesses convergence of item response accuracy parameters, item response time parameters, item variance and covariance matrix, person variance and covariance, higher-order latent structural parameters, higher-order latent ability, and latent speed; for the joint testlet-DINA model, PSRF assesses convergence of item response accuracy parameters, item response time parameters, item variance and covariance matrix, testlet variance and covariance matrix; person variance and covariance, higher-order latent structural parameters, higher-order latent ability, and latent speed; each panel includes plots of PSRF from 30 replications. In the below panel, PSRF of only 2 parameters (in two different replications) are larger than 1.1.
